# Development of an Agent-Based Model to Investigate the Impact of HIV Self-Testing Programs on Men Who Have Sex With Men in Atlanta and Seattle

**DOI:** 10.2196/publichealth.9357

**Published:** 2018-06-29

**Authors:** Wei Luo, David A Katz, Deven T Hamilton, Jennie McKenney, Samuel M Jenness, Steven M Goodreau, Joanne D Stekler, Eli S Rosenberg, Patrick S Sullivan, Susan Cassels

**Affiliations:** ^1^ Spatial Analysis Research Center School of Geographical Sciences and Urban Planning Arizona State University Tempe, AZ United States; ^2^ Department of Medicine University of Washington Seattle, WA United States; ^3^ Center for Studies in Demography and Ecology University of Washington Seattle, WA United States; ^4^ Department of Epidemiology Rollins School of Public Health Emory University Atlanta, GA United States; ^5^ Center for Studies in Demography and Ecology Department of Anthropology University of Washington Seattle, WA United States; ^6^ School of Public Health Epidemiology and Biostatistics Rensselaer Albany, NY United States; ^7^ Department of Geography University of California Santa Barbara Santa Barbara, CA United States

**Keywords:** HIV, men who have sex with men, pre-exposure prophylaxis

## Abstract

**Background:**

In the United States HIV epidemic, men who have sex with men (MSM) remain the most profoundly affected group. Prevention science is increasingly being organized around HIV testing as a launch point into an HIV prevention continuum for MSM who are not living with HIV and into an HIV care continuum for MSM who are living with HIV. An increasing HIV testing frequency among MSM might decrease future HIV infections by linking men who are living with HIV to antiretroviral care, resulting in viral suppression. Distributing HIV self-test (HIVST) kits is a strategy aimed at increasing HIV testing. Our previous modeling work suggests that the impact of HIV self-tests on transmission dynamics will depend not only on the frequency of tests and testers’ behaviors but also on the epidemiological and testing characteristics of the population.

**Objective:**

The objective of our study was to develop an agent-based model to inform public health strategies for promoting safe and effective HIV self-tests to decrease the HIV incidence among MSM in Atlanta, GA, and Seattle, WA, cities representing profoundly different epidemiological settings.

**Methods:**

We adapted and extended a network- and agent-based stochastic simulation model of HIV transmission dynamics that was developed and parameterized to investigate racial disparities in HIV prevalence among MSM in Atlanta. The extension comprised several activities: adding a new set of model parameters for Seattle MSM; adding new parameters for tester types (ie, regular, risk-based, opportunistic-only, or never testers); adding parameters for simplified pre-exposure prophylaxis uptake following negative results for HIV tests; and developing a conceptual framework for the ways in which the provision of HIV self-tests might change testing behaviors. We derived city-specific parameters from previous cohort and cross-sectional studies on MSM in Atlanta and Seattle. Each simulated population comprised 10,000 MSM and targeted HIV prevalences are equivalent to 28% and 11% in Atlanta and Seattle, respectively.

**Results:**

Previous studies provided sufficient data to estimate the model parameters representing nuanced HIV testing patterns and HIV self-test distribution. We calibrated the models to simulate the epidemics representing Atlanta and Seattle, including matching the expected stable HIV prevalence. The revised model facilitated the estimation of changes in 10-year HIV incidence based on counterfactual scenarios of HIV self-test distribution strategies and their impact on testing behaviors.

**Conclusions:**

We demonstrated that the extension of an existing agent-based HIV transmission model was sufficient to simulate the HIV epidemics among MSM in Atlanta and Seattle, to accommodate a more nuanced depiction of HIV testing behaviors than previous models, and to serve as a platform to investigate how HIV self-tests might impact testing and HIV transmission patterns among MSM in Atlanta and Seattle. In our future studies, we will use the model to test how different HIV self-test distribution strategies might affect HIV incidence among MSM.

## Introduction

To date, HIV testing remains the cornerstone of HIV prevention and care. Approximately 15% of 1.2 million individuals living with HIV in the United States are unaware of their infection [1,2], and some estimates suggest that these individuals account for almost one-third of all sexual transmissions in the United States [3]. HIV-positive individuals can be linked to HIV care and antiretroviral therapy (ART), which can improve individual health outcomes and decrease the likelihood of transmission [4]. In contrast, HIV-negative individuals can be linked to HIV prevention strategies, such as pre-exposure prophylaxis (PrEP) [5]. In the United States, men who have sex with men (MSM) constitute a high-risk group for acquiring HIV, and CDC recommends that MSM should undergo tests for HIV, at least, annually [6]. Despite this recommendation, only 56% of US MSM were reported to have undergone the tests in 2015 [7]. The low rate of testing has hindered linking the requisite number of MSM to treatment and prevention of HIV to bring down the rate of new infections below the effective reproductive number. Consequently, HIV diagnoses among young MSM, especially young black MSM in the United States, has been increasing [8].

In 2012, the United States Food and Drug Administration approved the first HIV self-test (HIVST), the OraQuick In-Home HIV Test (OraQuick; OraSure Technologies, Inc; Bethlehem, PA, USA). HIVSTs are the tests that can be entirely performed by the person undergoing the test for HIV, from specimen collection to reading and interpreting test results. One rationale for OraQuick’s approval was that the availability of self-testing—a testing modality that provides convenience, privacy, and anonymity—might decrease the number of people living with HIV who remain unaware of their HIV status [9]. HIVST promises to increase the frequency and coverage of testing in several ways. Self-testing offers a way to reach not only people who are in need of HIV testing but also those who are either not linked to medical care or hesitant to attend testing in community-based settings [10]. In addition, HIVST could provide opportunities to supplement current, clinic-based testing to elevate the testing frequency for MSM. However, HIVST using oral fluids has an extended window period, during which individuals recently infected with HIV might test falsely negative [11], which might incorrectly reassure people with recently acquired HIV infection; furthermore, such individuals are highly infectious [12]. Such people might delay treatment initiation and expose their uninfected partners to HIV. Moreover, they might postpone their next test, further delaying both diagnosis and treatment. Furthermore, people who test positive using HIVST might not be linked to care as frequently as those who test in more traditional settings, such as clinics.

Typically, HIVSTs have been found to be highly acceptable among MSM [13], and studies have established the feasibility and acceptability of various strategies for distributing HIVST kits among MSM [14], including social and sexual networks [15,16], geosocial networking apps [17], bathhouses [18], vending machines [19], vouchers [20], mass distribution at LGBTQ–focused events (lesbian, gay, bisexual, transgender, and queer, LGBTQ) [21], or Web-based strategies [22]. However, the use of HIVSTs in the general MSM population or outside of research studies remains partially investigated. Studies among Australian and Spanish MSM before local regulatory approval and among MSM receiving partner services for newly diagnosed HIV in New York City reported extremely low histories of prior HIVST use (<2.5%) [23-25]. Conversely, surveillance data from King County, WA, suggested that approximately 20% of MSM had used an HIVST by 2015 [21]. Empirical patterns of HIVST use and the extent to which men will supplement or replace clinic-based testing with HIVST will be elucidated in future studies. The objective of developing our present model was to investigate how different patterns of use might affect the HIV incidence and, thus, inform how to promote self-testing.

Our research groups have conducted prior modeling work in several areas related to HIV testing. Katz et al [26] developed a deterministic, continuous-time model of HIV transmission dynamics, suggesting that any replacement of clinic-based tests capable of detecting recent HIV infection with self-testing leads to an increased HIV prevalence among Seattle MSM, irrespective of the impact of self-tests on testing frequency. However, one limitation of this model was that it investigated replacing clinic-based tests only and did not consider other ways in which MSM may use HIVST [27]. Furthermore, there was lack of stochasticity in outcomes, infeasibility of parameterizing certain network structures, and exponential complexity with the linear addition of parameters. It would not be feasible to represent the level of heterogeneity in testing qualities, profiles of testers, and testing scenarios in a compartmental model, nor could the model account for specific sex acts and subsequent seroadaptive behavior based on the combination of test results. Khanna et al [28] reported the potential benefits of individualized HIV testing programs for decreasing HIV transmissions among MSM, and Delaney et al [29] used agent-based models to evaluate the potential value of focusing testing programs on MSM who had never been tested before. Both studies highlighted the potential benefits of HIV testing programs at the individual level to decrease the HIV prevalence among MSM; however, these agent-based models did not explicitly account for the use of HIVSTs or nuanced HIV testing behaviors.

Modeling offers an opportunity to process the available information from studies of self-testing, inform the design of self-testing interventions, compare how self-testing could be most effectively used in different epidemiological settings, and estimate the likely impact of scaling self-testing programs for MSM. Thus, we aimed to develop an agent-based model to investigate the potential impact of these tests on the HIV epidemic among MSM from two different epidemic settings in the United States—Atlanta, GA, and Seattle, WA. Approaches like ours are efficient, cost-effective, and complementary to community-level randomized controlled trials for assessing interventions like self-testing, especially in the setting of broad availability of self-tests and diverse programs, including self-tests being provided by health programs. Briefly, we aimed to develop a network-based mathematical model of HIV transmission dynamics in susceptible populations of MSM in Atlanta and Seattle.

## Methods

### Study Design

We adapted and extended a previously published, dynamic, stochastic network model of HIV transmission dynamics, which was originally designed to elucidate racial disparities in HIV prevalence among MSM in Atlanta [30]. The original model comprised key aspects of HIV transmission dynamics among MSM, including the occurrence of condomless anal intercourse (CAI) and related HIV transmission probabilities, sexual partnership characteristics (eg, main or casual partnerships), HIV testing frequency, and HIV care seeking. In the original model, MSM possessed fixed (eg, race and circumcision status) and dynamic (eg, age and infection status) attributes. In addition, HIV-infected men possessed additional dynamic attributes (eg, diagnosis status, treatment status, infection stage, and plasma HIV RNA level). Consistent with prior studies [31,32], sexual contact networks used separable temporal exponential random graph models (STERGMs) [33], a flexible statistical framework for simulating partnership formation and dissolution across networks [34], facilitating one to match data on the complex cross-sectional network structure, as well as reported relational durations. We implemented STERGMs in the R package suite *statnet* [35] and *EpiModel* [36]. The specific parameters for the sexual behavior developed by the domain and city are presented in Appendix Table A1.

### HIV Transmission and Progression

In the model, sexual contacts occurred on three networks, each of which shared the same set of nodes (people) but represented a different relationship type: main partners, casual partners, and one-time partners. Partnership formation depended on the following four predictors: partnership types (main, casual, or one-time partners), degree distribution (number of ongoing partners for each individual), age homophily (partners with similar age), and sexual role segregation (only those men who engaged in receptive anal sex could pair with men who engaged in exclusively insertive anal sex and vice versa). There was a constant relationship dissolution hazard for main and casual partnerships based on the median duration of each type (Multimedia Appendix 1, Table A1).

HIV progression followed both a natural trajectory of disease, in the absence of ART, and an ART-mediated trajectory [37]. In the absence of ART, HIV viral loads progressed in three stages: peak viremia during acute HIV infection (21 days); a set point viral load during the clinical latency (42 days); and a subsequent increase, resulting in AIDS and disease-induced mortality (728 days) [38]. In serodiscordant pairs, HIV transmission probabilities included those mediated by the viral load of the positive partner [39], condom use [40], the presence of the CCR5-Δ32 genetic allele [41,42], receptive versus insertive sexual position of the HIV-negative partner [31], and the circumcision status of an insertive HIV-negative partner [43]. After infection, men were assigned to three clinical care trajectories: those who never initiate treatment; those who initiate treatment and become partially suppressed; and those who initiate treatment and become entirely suppressed. The three trajectories exhibited different rates of infectiousness, HIV diagnosis, ART initiation, HIV viral suppression, and progression to AIDS and death to match empirical estimates of the prevalence of these states [44]. Furthermore, ART decreased the viral load and its associated transmission risk [4] and extended the life span [45].

### HIV Pre-Exposure Prophylaxis

We included PrEP use and adherence as additional features of the model. At any given time, 23.4% of HIV-negative MSM in the Seattle model and 11% of HIV-negative men in the Atlanta model were on PrEP according to Darcy Rao (oral communication, May 2017). If the proportion of HIV-negative MSM on PrEP decreased below the threshold coverage because of discontinuation or seroconversion, newly tested HIV-negative MSM were allowed to start PrEP. In addition, MSM on PrEP received a diagnostic HIV test at regular intervals (3 months). In fact, MSM who started PrEP were assigned a fixed adherence profile that reflected an average weekly dosage using data from the US PrEP Demo Project [46] weighted by race or ethnicity using methods from Jenness et al [32], which investigated the impact of the implementation of the US Centers for Disease Control and Prevention’s PrEP guidelines on the national HIV epidemic among MSM. For the Atlanta model, we assigned 21.1% of men as nonadherent (0 doses), 7.0% taking <2 doses/week, 10.0% taking 2-3 doses/week, and 61.9% taking ≥4 doses/week; for the Seattle model, we assigned 14.4% as nonadherent, 4.1% taking <2 doses/week, 5.3% taking 2-3 doses/week, and 76.2% taking ≥4 doses/week [32]. The probability of HIV acquisition per sex act was decreased according to the level of adherence. Little evidence for risk behavior changes among MSM while on PrEP was found in studies predating the development of this model [46-49]; thus, we focused on the PrEP coverage and adherence and did not model such behavior changes.

### HIV Testing: Clinic and Self-Testing

To investigate the possible impacts of HIV self-testing strategies on HIV epidemic dynamics in future models, we added more complex parameters describing clinic-based HIV testing behaviors to the original model to create a new baseline model. As existing patterns of the testing behavior vary among MSM, we conceptualized this baseline model with four tester types [50] (Table 1): (1) those who do not test in any setting (“never testers”); (2) those who test for HIV, but without a regular testing interval and without considering sexual episodes (eg, testing outreach at Pride festivals or when seeking medical care for a non-HIV or STD-related reason; “opportunistic-only testers”); (3) those who test for HIV regularly, irrespective of sexual behaviors (“regular testers”); and (4) those who test for HIV in response to a specific sexual episode (eg, CAI with a partner of unknown HIV serostatus; “risk-based testers”). Both regular and risk-based testers could also have an opportunity to become opportunistic testers and test outside of their regular testing interval or in the absence of a specific sexual episode, respectively, when opportunities were available. In addition, many public health agencies recommend different testing frequencies for MSM at higher versus lower risk for HIV acquisition [51-54], and evidence suggests that on an average, MSM at higher risk test more frequently [55-60]. Consequently, we stratified regular testers into high vs low anal intercourse (AI) frequency groups to allow for differential testing frequencies by risk.

The four categories of HIV tester types (Table 1) in the baseline model provide a framework to investigate the efficacy of different HIV self-testing scenarios for both Atlanta and Seattle. In the next stage of this research, we will implement two different self-testing intervention strategies: replacement testing and supplementary testing (Table 2). In the replacement testing scenarios, we will evaluate the proportion of HIV infections averted when HIVSTs replace clinic-based opportunistic tests, regular tests, and risk-based tests, respectively and simultaneously. In addition, we will assess the impact of replacing 25% and 50% of clinic-based tests with HIVSTs, assuming a 90-day window period (duration between when a person might have been exposed to HIV and when a test can give an accurate result) for self-tests [61].

We will also model the proportion of HIV infections averted when self-tests supplement clinic-based tests. In these scenarios, never testers and opportunistic-only testers take one or two supplementary HIVSTs randomly each year. Regular testers can supplement clinic-based tests with HIVSTs in two ways.

**Table 1:** HIV testing typology for the baseline model (clinic test only) of HIV transmission dynamics among men who have sex with men in Seattle and Atlanta.

Tester type	Baseline testing behavior	Opportunistic testing behavior
Never testers	None	None
Opportunistic-only testers	None	Likelihood of testing when presented with an opportunity to test (varies by the tester type)
Regular testers	Test interval varies by HIV risk category (defined by high vs low AI^a^ frequency)	
Risk-based testers	Testing likelihood and time to test varies by three types of events: (1) CAI^b^ in non‐main partnership, (2) CAI within known serodiscordant partnership, and (3) Acquisition of new main partner	




^a^AI: anal intercourse.

^b^CAI: condomless anal intercourse.

**Table 2:** Scenarios for modeling uptake of HIV self-testing among men who have sex with men: replacement versus supplementary testing (to be compared with the baseline model).

Tester type	Replacement scenarios	Supplementation scenarios
Never testers	N/A^a^	1 test per year 2 tests per year
Opportunistic-only testers	25% replacement 50% replacement	1 additional test per year 2 additional tests per year
Risk-based testers	25% replacement 50% replacement	10% additional probability of testing after risk event 20% additional probability
Regular testers	25% replacement 50% replacement	1 additional test per year at random time Decrease the intertest interval by adding 1 test per year

^a^N/A: not applicable.

First, they can supplement clinic tests with one additional HIVST randomly during a year. Second, they can test more frequently (ie, shorter intertest interval) using both clinic tests and self-tests. For example, if a regular tester tests three times a year in a clinic, his intertest interval would be 120 days; if he supplements this with one self-test per year, his intertest interval would be 90 days, and he would have a 25% chance that each test would be a self-test. Risk-based testers supplement their clinic-based tests by increasing the probability of risk-based tests by 10% and 20%. For example, if a risk tester has a 40% chance of taking a clinic-based test after a risk event and self-testing increases the probability of risk-based testing by 10%, his likelihood of testing becomes 50% with a 40% chance of taking a clinic-based test and a 10% chance of self-testing.

## Results

### Data Parameterization

We hypothesized that the impact of HIVST on MSM will differ in cities with different underlying patterns of HIV testing, access to HIV prevention and care, and epidemiological characteristics of MSM. King County (home to Seattle) is one of the first jurisdictions to reach the World Health Organization’s 90-90-90 goals [62] and has witnessed a rapid scale-up of PrEP [63]. HIV prevalence among MSM in Seattle is estimated to be 11% according to Dr Susan Buskin (personal communication, October 2017), and the rate of new HIV diagnoses has been declining over the last decade [64]. In contrast, the HIV prevalence among MSM in Atlanta is estimated to be much higher at 28% [65], with fewer MSM aware of their HIV infection status [66], exhibiting less access to PrEP and a more diverse population with significant disparities in HIV by race [67]. The data sources for parameterizing our model for each city are presented in Table 3, with additional detail in the Appendix Table A1. As the original model was structured to assess racial differences in the incidence, where race-stratified estimates were available, we used those estimates to create race-weighted composite estimates for the overall population in each city. Both HIV testing and network-based behavioral data were collected in a prospective cohort study [65] and an egocentric sexual network study [68] of MSM in Atlanta and cross-sectional surveys of MSM in Seattle [55,69].

We estimated parameters to characterize HIV testing behaviors in both Atlanta and Seattle, including the proportions of the four tester types (Table 4) and mean test intervals and test likelihood for opportunistic-only, regular, and risk-based testers (Table 5). Of note, these HIV testing parameters only apply to MSM who are not on PrEP (MSM on PrEP received a diagnostic HIV test at regular 3-month intervals). For Seattle, the proportions of the four tester types, the mean intervals between regular tests stratified by the frequency of AI, and the likelihood that opportunistic testers will seize a testing opportunity were estimated using data obtained from 361 HIV-negative and unknown-status MSM aged 18-39 years who participated in the Seattle Pride Survey [63] in 2013 and 2014 and reported living in Washington State. The Pride Survey is an annual convenience sample of self-identified MSM enrolled from along the Seattle Pride Parade route to complete a self-administered survey to monitor HIV risk behaviors and the uptake of treatment and prevention interventions. The 2013 and 2014 surveys included the following question: “How many HIV tests did you have in the last two years?” To calculate the mean interval in days between regular tests, we divided 2 years (730.5 days) by the mean number of HIV tests reported in the last 2 years among the participants reporting their last test as a “routine test,” stratified by the number of AI partners in the last year (0-2 vs 3+ partners). In addition, we calculated the likelihood that opportunistic-only testers will seize a testing opportunity by dividing the mean number of HIV tests reported in the last 2 years by the expected number of opportunities to test in a 2-year period (4 opportunities: 2 each year, based on expert opinion) among participants who reported the reason for their last test as something other than risk-based or routine.

For MSM in Atlanta, the proportions of the four tester types were estimated using data from the Man Project [68] and InvolveMENt [65]. The Man Project was a cross-sectional, chain-referral sexual network study among 314 MSM; however, InvolveMENt was a prospective cohort study among 803 black and white non-Hispanic MSM aged 18-39 years. Participants for both the studies completed self-administered computer-based questionnaires that assessed demographics as well as risk and prevention behaviors for both themselves and their most recent sex partners (up to the last 10 partners in the last 12 months for the Man Project and the last 5 partners in the previous 6 months for InvolveMENt). We evaluated the mean interval in days between regular tests using data from the PUMA Survey [70], an internet-based survey of MSM in the United States conducted from November 30, 2010 to December 19, 2010 (*n*=1251). Men who reported regularly testing for HIV were asked how often they tested (in months). Then, we calculated the mean number of months between regular tests, but unlike for the parameters of MSM in Seattle, we were unable to stratify this interval by the number of AI partners. Furthermore, we calculated the likelihood that opportunistic-only testers seized an opportunity from data collected in the Annual American Men’s Internet Survey of Behaviors of Men who have Sex with Men in the United States (AMIS) [7,69,71]. AMIS is an annual cross-sectional behavioral survey of MSM in the United States in which participants complete a Web-based survey that includes questions in the following domains: demographics, sexual behavior, HIV testing history, drug and alcohol use, and HIV prevention services exposure. As with the Seattle data, the likelihood was calculated among participants who self-reported living in Georgia and reported testing for reasons other than risk or routine by dividing the mean number of HIV tests reported among them in the last 2 years by the expected number of opportunities in that period.

Where local data for parameterization were unavailable for both cities, we used estimates from national Web-based surveys conducted by research teams involving members of our group. The likelihood of seizing a testing opportunity among regular and risk-based testers was estimated by calculating the proportion of individuals who reported currently testing on a regular schedule and reported that their last test was in response to an opportunity using data from the Internet Ethics and Incentives Study [7,37,69]. Specifically, to parameterize this model, we added questions to the 2016 cycle of AMIS [7,37,69] to ascertain the likelihood of testing following a risk event and the time from the risk event to test. Respondents were asked whether they tested in response to each of the following events: the last time they had CAI with a nonmain partner, the last time they had CAI with a serodiscordant partner, and the last time they started a new relationship. In addition, we calculated the likelihood of testing following each event as the proportion of individuals who reported testing in response to such an event among those who reported the event. Subsequently, men who reported that they last tested in response to CAI with a nonmain partner, CAI with a serodiscordant partner, or starting a new relationship were asked how many weeks following the event they tested. The time from event to test was calculated as the mean number of weeks for each type of event separately.

### Model Calibration

**Figure 1:**
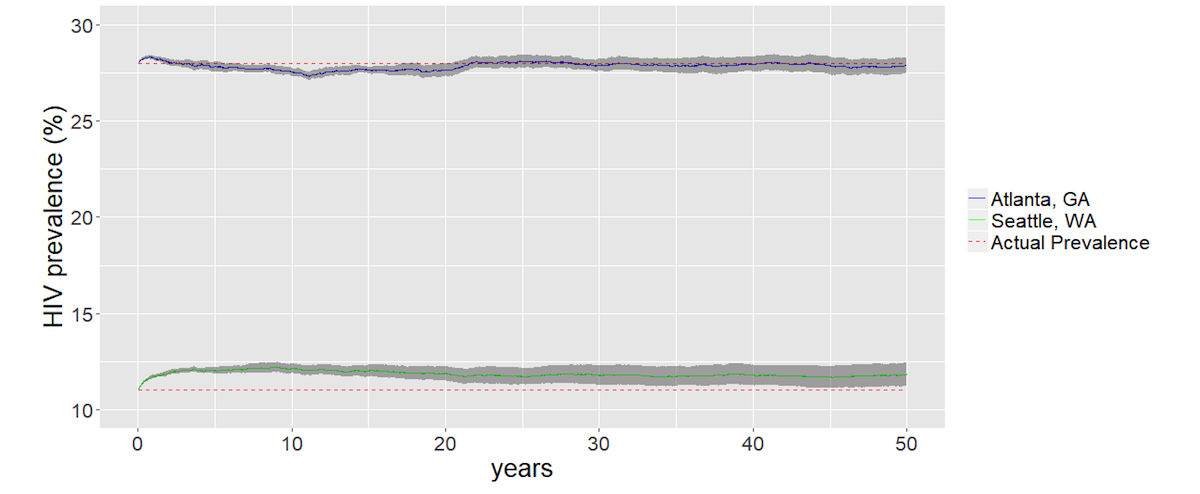
Simulated HIV prevalence plots to produce baseline epidemics for Atlanta, GA, and Seattle, WA, with 95% CIs (gray band). The actual HIV prevalence among the men who have sex with men population was 28% in Atlanta and 11% in Seattle.

Using the above parameters, we simulated the sexual, vital, and epidemiological dynamics of HIV transmission among 10,000 MSM for 50 years using Seattle- and Atlanta-specific parameterizations and, at equilibrium, reproduced the epidemiological and demographic outcomes observed in Seattle and Atlanta, respectively. In addition, we calibrated the baseline models by varying the rate of AI within partnerships to closely match the estimates of HIV prevalence that were 28% in Atlanta and 11% in Seattle among MSM (Figure 1). We used a multiplier of 1.5 in Atlanta and 3.45 in Seattle to adjust the AI rate, which resulted in simulated epidemics equilibrating at 27.24% (95% CI: 27.79-28.35) in Atlanta and 11.55% (95% CI: 11.36-11.73) in Seattle. To select the multiplier, we varied the AI multiplier by 0.01 within preselected ranges of 1.45-1.55 and 3.4-3.5 for Atlanta and Seattle, respectively. Then, we identified the value that resulted in an average equilibrium HIV prevalence (from 100 simulations for each value for 50 years each) closest to the target estimates.

The next steps in our research will be to introduce each self-test scenario into the simulated populations and project epidemic outcomes over a 10-year time horizon. We will further investigate how different test sensitivities and detection windows affect the impact of HIVST.

**Table 3:** Data sources for parameterization of a model of HIV transmission dynamics among MSM in Atlanta, GA, and Seattle, WA.

Parameters	Atlanta Data Sources	Seattle Data Sources
Testing and treatment	InvolveMENt [65,72-74]	Medical Monitoring Project [75,76], HIV Surveillance [62,77]
Tester type	AMIS^a^ [7,69,71], Man Project [68], InvolveMENt [65]	Seattle Pride Survey [63]
Mean interval between tests among regular testers	PUMA^b^ Survey [70]	Seattle Pride Survey [63]
Likelihood of seizing the testing opportunity for risk-based, regular, and opportunity-only testers	Internet Ethics and Incentives Study [78], American Men's Internet Survey [7,69,71]	Internet Ethics and Incentives Study [78], Seattle Pride Survey [63]
Risk-based testing: the likelihood of testing after event and time from event to test	American Men's Internet Survey [7,69,71]	American Men's Internet Survey [7,69,71]
Sexual behavior (ie, versatility, condom use, disclosure); sexual network attributes	Man Project [68], InvolveMENt [65]	Mobile Study (S. Cassels, Personal Communication, November 2017)
Prevalence of circumcision	InvolveMENt [65]	National HIV Behavioral Surveillance [66,79,80]
PrEP coverage	PUMA Survey [70]	Washington HIV Prevention Project
Expected coital frequency within partnerships	PUMA Survey [70]	Washington HIV Prevention Project
Racial or ethnic distribution of MSM for weighting parameters estimates	Goodreau et al [30]	King County Population Estimates [81]
CCR5-Δ32 prevalence	Marmor et al [82]	Zimmerman et al [41] and Marmor et al [82]

^a^AMIS: Annual American Men’s Internet Survey of Behaviors of Men who have Sex with Men in the United States.

^b^PUMA: Prevention Umbrella for MSM in the Americas.

**Table 4:** Estimated proportions of four tester types in Atlanta and Seattle.

Tester type	Atlanta	Seattle
Never testers	3.5%	2.5%
Opportunistic-only testers	37.0%	13.8%
Regular testers	44.0%	64.9%
Risk-based testers	15.5%	18.8%

**Table 5:** Mean test intervals and test likelihood for opportunistic-only, regular, and risk-based testers in both Atlanta and Seattle.

Tester Type		Atlanta	Seattle
**Opportunistic-only testers**			
	Interval between opportunities to take an opportunistic test	183 days	183 days
	Likelihood of seizing testing opportunity: Opportunistic-only testers	0.629	0.764
**Regular testers**			
	Interval between regular tests for high AI frequency group	224 days	151 days
	Interval between regular tests for low AI frequency group	224 days	372 days
	Likelihood of seizing testing opportunity: Regular tester	0.095	0.095
**Risk-based testers**			
	Likelihood of testing after event: CAI in non-main partnership	0.339	0.359
	Likelihood of testing after event: CAI within known serodiscordant partnership	0.520	0.538
	Likelihood of testing after event: Acquisition of new main partner	0.349	0.375
	Time from event to test: CAI in non-main partnership	39.2 days	39.2 days
	Time from event to test: CAI within known serodiscordant partnership	43.4 days	43.4 days
	Time from event to test: Acquisition of new main partner	56.7 days	56.7 days
	Likelihood of seizing testing opportunity: Risk-based tester	0.095	0.095

## Discussion

This study adapted our previous dynamic, network-based epidemic model of HIV transmission dynamics [30] and conceptualized and implemented a baseline model as a platform for investigating different HIVST intervention strategies at the individual level. These dynamic network models are ideal for investigating the effects of complex, interacting interventions along structured and evolving contact networks [31]. Our primary improvement was adding four different types of testers to this model (never testers, opportunistic-only testers, regular testers, and risk-based testers) and deriving estimates of prevalence as well as patterns of testing behaviors for each tester type. It will enable us to better reflect the range of testing behaviors among MSM than other models, which usually assume that all men test in the same manner, and will enable us to assess the potential impact of targeting self-testing to specific types of testers. In addition, we estimated the parameters related to HIV behaviors among MSM in Atlanta and Seattle to compare the impact of different epidemic settings on HIVST intervention strategies. Our enhanced model will be used to examine self-testing intervention strategies to determine the most effective ways to promote HIVST for MSM in the United States. Furthermore, our models offer great promise to account for novel testing strategies and to inform public health approaches for the promotion of safe and effective HIV self-testing strategies in two divergent settings, Seattle and Atlanta.

As with all models, ours has several limitations. First, to match the observed HIV prevalence in Seattle and Atlanta, our models required calibration for one behavioral parameter, the frequency of AI within partnerships. Not all factors that contribute to the differential HIV transmission dynamics could be included in this model; thus, the variation in the frequency of AI within partnerships could also represent the variation in the processes not included in the model. For example, we adopted a simplistic approach to PrEP use in our model. PrEP indications, uptake, and use over time are complex behaviors, and individuals who initiate PrEP might be nonrandomly associated with certain tester types. However, we modeled PrEP use as a consistent coverage fraction and did not have detailed data to parameterize how PrEP use interacted with our newly parameterized testing categories. Our models were based on data collected from numerous data sources, rather than one comprehensive, representative source for each city, and in some cases, local data sources were unavailable; thus, we had to rely on national surveys that are unlikely to be representative of either city. Second, testing parameters relied primarily on self-reported behaviors. Thus, the findings from the model will be considered as hypothetical scenarios to illustrate how similar approaches to HIV testing, self-testing in particular, can affect HIV transmission dynamics in various ways in different epidemic settings. Furthermore, our model should not be used as a prediction tool to assess the trajectory of the epidemic for specific subpopulations. Finally, the testing categories are static in the model. Hence, further investigations are warranted to elucidate how individual testing behavior varies over the life span.

HIVST provides a new opportunity to reach out to people who are in need of HIV testing. Understanding how to maximize the potential of HIV self-testing—and limit its risks—in the current landscape of high-sensitivity and high-throughput HIV testing is important. However, evaluations of self-testing programs have been limited in that it is difficult to assess who might be using the kits, what the results of the tests are, and, thus, what impact the self-tests might have on the epidemic. We executed a new modeling project to focus on the potential roles of self-tests in increasing HIV testing and decreasing the HIV incidence among MSM and to develop a model that would facilitate the assessment of how local epidemic characteristics might affect the impact of self-testing programs. The objectives were attained by developing parallel models for Seattle and Atlanta, which are different in their epidemics among MSM regarding testing behaviors; access to HIV testing, prevention, and care; and demographic and epidemic characteristics [83]. The finalization of this protocol will enable us to examine the population-level effects of HIVST; test different HIVST intervention strategies, such as replacement and supplementary testing, and mass and targeted distributions; and determine populations and settings where HIVST might have the greatest impact [84]. Overall, this study represents one more step forward in unfolding the process of improving the realism of epidemic models, and parameterizing them using rich local data, to best determine the optimal strategies for promoting population health in different communities.

## Appendix

Multimedia Appendix 1 The specific parameters for sexual behavior developed by domain and city.

## Abbreviations

AI: anal intercourse
ART: antiretroviral therapy
CAI: condomless anal intercourse
CDC: Centers for Disease Control and Prevention
HIVST: HIV self-test
MSM: men who have sex with men
PrEP: pre-exposure prophylaxis
PUMA: Prevention Umbrella for MSM in the Americas

## References

[1] Hall HI, An Q, Tang T, Song R, Chen M, Green T, Kang J, Centers FDC( (2015). Prevalence of Diagnosed and Undiagnosed HIV Infection--United States, 2008-2012. MMWR Morb Mortal Wkly Rep.

[2] Centers for Disease Control Prevention (2014). Monitoring selected national HIV prevention and care objectives by using HIV surveillance data-United States and 6 dependent areas-2012.

[3] Skarbinski J, Rosenberg E, Paz-Bailey G, Hall HI, Rose CE, Viall AH, Fagan JL, Lansky A, Mermin JH (2015). Human immunodeficiency virus transmission at each step of the care continuum in the United States. JAMA Intern Med.

[4] Cohen MS, Chen YQ, McCauley M, Gamble T, Hosseinipour MC, Kumarasamy N, Hakim JG, Kumwenda J, Grinsztejn B, Pilotto JHS, Godbole SV, Mehendale S, Chariyalertsak S, Santos BR, Mayer KH, Hoffman IF, Eshleman SH, Piwowar-Manning E, Wang L, Makhema J, Mills LA, de BG, Sanne I, Eron J, Gallant J, Havlir D, Swindells S, Ribaudo H, Elharrar V, Burns D, Taha TE, Nielsen-Saines K, Celentano D, Essex M, Fleming TR (2011). Prevention of HIV-1 infection with early antiretroviral therapy. N Engl J Med.

[5] Cohen MS, Chen YQ, McCauley M, Gamble T, Hosseinipour MC, Kumarasamy N, Hakim JG, Kumwenda J, Grinsztejn B, Pilotto JHS, Godbole SV, Mehendale S, Chariyalertsak S, Santos BR, Mayer KH, Hoffman IF, Eshleman SH, Piwowar-Manning E, Wang L, Makhema J, Mills LA, de BG, Sanne I, Eron J, Gallant J, Havlir D, Swindells S, Ribaudo H, Elharrar V, Burns D, Taha TE, Nielsen-Saines K, Celentano D, Essex M, Fleming TR (2011). Prevention of HIV-1 infection with early antiretroviral therapy. N Engl J Med.

[6] Centers for Disease Control and Prevention, 2017 (2017). Recommendations for HIV Screening of Gay, Bisexual, and Other Men Who Have Sex with Men – United States.

[7] Zlotorzynska M, Sullivan P, Sanchez T (2017). The Annual American Men's Internet Survey of Behaviors of Men Who Have Sex With Men in the United States: 2015 Key Indicators Report. JMIR Public Health Surveill.

[8] Rosenberg ES, Grey JA, Sanchez TH, Sullivan PS (2016). Rates of Prevalent HIV Infection, Prevalent Diagnoses, and New Diagnoses Among Men Who Have Sex With Men in US States, Metropolitan Statistical Areas, and Counties, 2012-2013. JMIR Public Health Surveill.

[9] U.S. Food and Drug Administration (2018). Available: Accessed 15 January.

[10] Walensky RP, Paltiel AD (2006). Rapid HIV testing at home: does it solve a problem or create one?. Ann Intern Med.

[11] Stekler JD, O'Neal JD, Lane A, Swanson F, Maenza J, Stevens CE, Coombs RW, Dragavon JA, Swenson PD, Golden MR, Branson BM (2013). Relative accuracy of serum, whole blood, and oral fluid HIV tests among Seattle men who have sex with men. J Clin Virol.

[12] Wawer MJ, Gray RH, Sewankambo NK, Serwadda D, Li X, Laeyendecker O, Kiwanuka N, Kigozi G, Kiddugavu M, Lutalo T, Nalugoda F, Wabwire-Mangen F, Meehan MP, Quinn TC (2005). Rates of HIV-1 transmission per coital act, by stage of HIV-1 infection, in Rakai, Uganda. J Infect Dis.

[13] Figueroa C, Johnson C, Verster A, Baggaley R (2015). Attitudes and acceptability on HIV self-testing among key populations: a literature review. AIDS Behav.

[14] Estem KS, Catania J, Klausner JD (2016). HIV self-testing: a review of current implementation and fidelity. Curr HIV/AIDS Rep.

[15] Carballo-Diéguez A, Frasca T, Balan I, Ibitoye M, Dolezal C (2012). Use of a rapid HIV home test prevents HIV exposure in a high risk sample of men who have sex with men. AIDS Behav.

[16] Lippman SA, Lane T, Rabede O, Gilmore H, Chen Y, Mlotshwa N, Maleke K, Marr A, McIntyre JA (2018). High acceptability and increased HIV-testing frequency after introduction of HIV self-testing and network distribution among South African MSM. J Acquir Immune Defic Syndr.

[17] Huang E, Marlin RW, Young SD, Medline A, Klausner JD (2016). Using Grindr, a smartphone social-networking application, to increase HIV self-testing among black and Latino men who have sex with men in Los Angeles, 2014. AIDS Educ Prev.

[18] Woods WJ, Lippman SA, Agnew E, Carroll S, Binson D (2016). Bathhouse distribution of HIV self-testing kits reaches diverse, high-risk population. AIDS Care.

[19] Young SD, Klausner J, Fynn R, Bolan R (2014). Electronic vending machines for dispensing rapid HIV self-testing kits: a case study. AIDS Care.

[20] Marlin RW, Young SD, Bristow CC, Wilson G, Rodriguez J, Ortiz J, Mathew R, Klausner JD (2014). Piloting an HIV self-test kit voucher program to raise serostatus awareness of high-risk African Americans, Los Angeles. BMC Public Health.

[21] Katz D, Bennett A, Dombrowski J, Hood J, Buskin S, Golden M 2015 National HIV Prevention Conference, Atlanta, GA.

[22] Edelstein Z, Salcuni P, Tsoi B CROI 2017; Seattle, WA.

[23] Koutentakis K, Rosales-Statkus ME, Hoyos J, Fernández-Balbuena S, Ruiz M, Agustí C, de LFL, Belza MJ, Madrid HIV self-testing group (2016). Knowledge and use of unauthorized HIV self-test kits among men who have sex with men in Spain, following approval of an over-the-counter self-test in the U.S: a cross-sectional study. BMC Public Health.

[24] Prestage G, Zablotska I, Bavinton B, Grulich A, Keen P, Murphy D, Brown G, Bradley J, Holt M, Guy R (2016). Previous and future use of HIV self-testing: a survey of Australian gay and bisexual men. Sex Health.

[25] Udeagu CN, Shah S, Molochevski M (2017). Men who have sex with men seek timely human immunodeficiency virus confirmation and care after rapid human immunodeficiency virus self-test: data from partner services program, New York City. Sex Transm Dis.

[26] Katz DA, Cassels SL, Stekler JD (2014). Replacing clinic-based tests with home-use tests may increase HIV prevalence among Seattle men who have sex with men: evidence from a mathematical model. Sex Transm Dis.

[27] Jamil MS, Prestage G, Fairley CK, Grulich AE, Smith KS, Chen M, Holt M, McNulty AM, Bavinton BR, Conway DP, Wand H, Keen P, Bradley J, Kolstee J, Batrouney C, Russell D, Law M, Kaldor JM, Guy RJ (2017). Effect of availability of HIV self-testing on HIV testing frequency in gay and bisexual men at high risk of infection (FORTH): a waiting-list randomised controlled trial. Lancet HIV.

[28] Khanna A, Goodreau SM, Wohlfeiler D, Daar E, Little S, Gorbach PM (2015). Individualized diagnosis interventions can add significant effectiveness in reducing human immunodeficiency virus incidence among men who have sex with men: insights from Southern California. Ann Epidemiol.

[29] Delaney KP, Rosenberg ES, Kramer MR, Waller LA, Sullivan PS (2015). Optimizing human immunodeficiency virus testing interventions for men who have sex with men in the United States: a modeling study. Open Forum Infect Dis.

[30] Goodreau SM, Rosenberg ES, Jenness SM, Luisi N, Stansfield SE, Millett GA, Sullivan PS (2017). Sources of racial disparities in HIV prevalence in men who have sex with men in Atlanta, GA, USA: a modelling study. Lancet HIV.

[31] Goodreau SM, Carnegie NB, Vittinghoff E, Lama JR, Sanchez J, Grinsztejn B, Koblin BA, Mayer KH, Buchbinder SP (2012). What drives the US and Peruvian HIV epidemics in men who have sex with men (MSM)?. PLoS One.

[32] Jenness SM, Goodreau SM, Rosenberg E, Beylerian EN, Hoover KW, Smith DK, Sullivan P (2016). Impact of the Centers for Disease Control's HIV Preexposure Prophylaxis Guidelines for men who have sex with men in the United States. J Infect Dis.

[33] Krivitsky PN, Morris M (2017). Inference for social network models from egocentrically sampled data, with application to understanding persistent racial disparities in HIV prevalence in the US. Ann. Appl. Stat.

[34] Krivitsky PN, Handcock MS (2014). A separable model for dynamic networks. J R Stat Soc Series B Stat Methodol.

[35] Handcock Mark S, Hunter David R, Butts Carter T, Goodreau Steven M, Morris Martina (2008). statnet: Software Tools for the Representation, Visualization, Analysis and Simulation of Network Data. J Stat Softw.

[36] Jenness Samuel M, Goodreau Steven M, Morris Martina (2018). EpiModel: An R Package for Mathematical Modeling of Infectious Disease over Networks. J Stat Softw.

[37] Carnegie NB, Goodreau SM, Liu A, Vittinghoff E, Sanchez J, Lama JR, Buchbinder S (2015). Targeting pre-exposure prophylaxis among men who have sex with men in the United States and Peru: partnership types, contact rates, and sexual role. J Acquir Immune Defic Syndr.

[38] Pilcher CD, Price MA, Hoffman IF, Galvin S, Martinson FEA, Kazembe PN, Eron JJ, Miller WC, Fiscus SA, Cohen MS (2004). Frequent detection of acute primary HIV infection in men in Malawi. AIDS.

[39] Hughes JP, Baeten JM, Lingappa JR, Magaret AS, Wald A, de BG, Kiarie J, Inambao M, Kilembe W, Farquhar C, Celum C, Partners in Prevention HSV/HIV Transmission Study Team (2012). Determinants of per-coital-act HIV-1 infectivity among African HIV-1-serodiscordant couples. J Infect Dis.

[40] Weller S, Davis K (2002). Condom effectiveness in reducing heterosexual HIV transmission. Cochrane Database Syst Rev.

[41] Zimmerman PA, Buckler-White A, Alkhatib G, Spalding T, Kubofcik J, Combadiere C, Weissman D, Cohen O, Rubbert A, Lam G, Vaccarezza M, Kennedy PE, Kumaraswami V, Giorgi JV, Detels R, Hunter J, Chopek M, Berger EA, Fauci AS, Nutman TB, Murphy PM (1997). Inherited resistance to HIV-1 conferred by an inactivating mutation in CC chemokine receptor 5: studies in populations with contrasting clinical phenotypes, defined racial background, and quantified risk. Mol Med.

[42] Marmor M, Sheppard HW, Donnell D, Bozeman S, Celum C, Buchbinder S, Koblin B, Seage GR, HIV Network for Prevention Trials Vaccine Preparedness Protocol Team (2001). Homozygous and heterozygous CCR5-Delta32 genotypes are associated with resistance to HIV infection. J Acquir Immune Defic Syndr.

[43] Wiysonge CS, Kongnyuy EJ, Shey M, Muula AS, Navti OB, Akl EA, Lo Y (2011). Male circumcision for prevention of homosexual acquisition of HIV in men. Cochrane Database Syst Rev.

[44] Sullivan PS, Rosenberg ES, Sanchez TH, Kelley CF, Luisi N, Cooper HL, Diclemente RJ, Wingood GM, Frew PM, Salazar LF, Del RC, Mulligan MJ, Peterson JL (2015). Explaining racial disparities in HIV incidence in black and white men who have sex with men in Atlanta, GA: a prospective observational cohort study. Ann Epidemiol.

[45] Goodreau SM, Carnegie NB, Vittinghoff E, Lama JR, Fuchs JD, Sanchez J, Buchbinder SP (2014). Can male circumcision have an impact on the HIV epidemic in men who have sex with men?. PLoS One.

[46] Liu AY, Cohen SE, Vittinghoff E, Anderson PL, Doblecki-Lewis S, Bacon O, Chege W, Postle BS, Matheson T, Amico KR, Liegler T, Rawlings MK, Trainor N, Blue RW, Estrada Y, Coleman ME, Cardenas G, Feaster DJ, Grant R, Philip SS, Elion R, Buchbinder S, Kolber MA (2016). Preexposure prophylaxis for HIV infection integrated with municipal- and community-based sexual health services. JAMA Intern Med.

[47] Grant RM, Anderson PL, McMahan V, Liu A, Amico KR, Mehrotra M, Hosek S, Mosquera C, Casapia M, Montoya O, Buchbinder S, Veloso VG, Mayer K, Chariyalertsak S, Bekker L, Kallas EG, Schechter M, Guanira J, Bushman L, Burns DN, Rooney JF, Glidden DV, iPrEx ST (2014). Uptake of pre-exposure prophylaxis, sexual practices, and HIV incidence in men and transgender women who have sex with men: a cohort study. Lancet Infect Dis.

[48] McCormack S, Dunn DT, Desai M, Dolling DI, Gafos M, Gilson R, Sullivan AK, Clarke A, Reeves I, Schembri G, Mackie N, Bowman C, Lacey CJ, Apea V, Brady M, Fox J, Taylor S, Antonucci S, Khoo SH, Rooney J, Nardone A, Fisher M, McOwan A, Phillips AN, Johnson AM, Gazzard B, Gill ON (2016). Pre-exposure prophylaxis to prevent the acquisition of HIV-1 infection (PROUD): effectiveness results from the pilot phase of a pragmatic open-label randomised trial. Lancet.

[49] Marcus JL, Glidden DV, Mayer KH, Liu AY, Buchbinder SP, Amico KR, McMahan V, Kallas EG, Montoya-Herrera O, Pilotto J, Grant RM (2013). No evidence of sexual risk compensation in the iPrEx trial of daily oral HIV preexposure prophylaxis. PLoS One.

[50] Hussen SA, Stephenson R, del RC, Wilton L, Wallace J, Wheeler D, HPTN 061 Protocol Team (2013). HIV testing patterns among black men who have sex with men: a qualitative typology. PLoS One.

[51] British HA, British AOSH (2008). UK National Guidelines for HIV Testing.

[52] Sexually Transmissible Infections in Gay Men Action Group (2014). Australian Sexually Transmitted Infection & HIV Testing Guidelines.

[53] Public Health - Seattle & King County (2017). Available at: Accessed 11 Aug.

[54] Workowski KA, Berman SM (2007). Centers for Disease Control and Prevention sexually transmitted diseases treatment guidelines. Clin Infect Dis.

[55] Katz DA, Dombrowski JC, Swanson F, Buskin SE, Golden MR, Stekler JD (2013). HIV intertest interval among MSM in King County, Washington. Sex Transm Infect.

[56] MacKellar DA, Valleroy LA, Anderson JE, Behel S, Secura GM, Bingham T, Celentano DD, Koblin BA, LaLota M, Shehan D, Thiede H, Torian LV, Janssen RS (2006). Recent HIV testing among young men who have sex with men: correlates, contexts, and HIV seroconversion. Sex Transm Dis.

[57] Manning SE, Thorpe LE, Ramaswamy C, Hajat A, Marx MA, Karpati AM, Mostashari F, Pfeiffer MR, Nash D (2007). Estimation of HIV prevalence, risk factors, and testing frequency among sexually active men who have sex with men, aged 18-64 years--New York City, 2002. J Urban Health.

[58] Visser M, Heijne JCM, Hogewoning AA, van AF (2017). Frequency and determinants of consistent STI/HIV testing among men who have sex with men testing at STI outpatient clinics in the Netherlands: a longitudinal study. Sex Transm Infect.

[59] Johnson BA, McKenney J, Ricca AV, Rosenberg ES, Liu C, Sharma A, Sullivan PS (2016). Risk factors associated with repeated HIV testing among internet-using men who have sex with men. AIDS Educ Prev.

[60] Hoenigl M, Anderson CM, Green N, Mehta SR, Smith DM, Little SJ (2015). Repeat HIV-testing is associated with an increase in behavioral risk among men who have sex with men: a cohort study. BMC Med.

[61] Stekler JD, Ure G, O'Neal JD, Lane A, Swanson F, Maenza J, Stevens C, Coombs RW, Dragavon J, Swenson PD, Golden MR (2016). Performance of Determine Combo and other point-of-care HIV tests among Seattle MSM. J Clin Virol.

[62] HIV/ AIDS Epidemiology Unit, Public Health - Seattle & King Countythe Infectious Disease Assessment Unit, Washington State Department of Health HIV/AIDS Epidemiology Report 2016, Volume.

[63] Hood JE, Buskin SE, Dombrowski JC, Kern DA, Barash EA, Katzi DA, Golden MR (2016). Dramatic increase in preexposure prophylaxis use among MSM in Washington state. AIDS.

[64] Golden MR, Bennett AB, Dombrowski JC, Buskin SE (2016). Achieving the goals of the National HIV/AIDS strategy: declining HIV diagnoses, improving clinical outcomes, and diminishing racial/ethnic disparities in King County, WA (2004-2013). Sex Transm Dis.

[65] Sullivan PS, Peterson J, Rosenberg ES, Kelley CF, Cooper H, Vaughan A, Salazar LF, Frew P, Wingood G, Diclemente R, del RC, Mulligan M, Sanchez TH (2014). Understanding racial HIV/STI disparities in black and white men who have sex with men: a multilevel approach. PLoS One.

[66] Centers for Disease Control and Prevention HIV Surveillance Special Report 15.

[67] Giustini Dean OA Librarian.

[68] Hernández-Romieu AC, Sullivan PS, Rothenberg R, Grey J, Luisi N, Kelley CF, Rosenberg ES (2015). Heterogeneity of HIV prevalence among the sexual networks of black and white men who have sex with men in Atlanta: illuminating a mechanism for increased HIV risk for young black men who have sex with men. Sex Transm Dis.

[69] Sanchez T, Zlotorzynska M, Sineath C, Kahle E, Sullivan P (2016). The Annual American Men's Internet Survey of Behaviors of Men Who have Sex with Men in the United States: 2014 key indicators report. JMIR Public Health Surveill.

[70] Volk Jonathan E, Liu Albert, Vittinghoff Eric, Irvin Risha, Kroboth Elizabeth, Krakower Douglas, Mimiaga Matthew J, Mayer Kenneth H, Sullivan Patrick S, Buchbinder Susan P (2012). Sexual frequency and planning among at-risk men who have sex with men in the United States: implications for event-based intermittent pre-exposure prophylaxis. J Acquir Immune Defic Syndr.

[71] Sanchez TH, Sineath RC, Kahle EM, Tregear SJ, Sullivan PS (2015). The Annual American Men's Internet Survey of Behaviors of Men Who Have Sex With Men in the United States: protocol and key indicators report 2013. JMIR Public Health Surveill.

[72] Rosenberg ES, Millett GA, Sullivan PS, Del RC, Curran JW (2014). Understanding the HIV disparities between black and white men who have sex with men in the USA using the HIV care continuum: a modeling study. Lancet HIV.

[73] Fiebig EW, Wright DJ, Rawal BD, Garrett PE, Schumacher RT, Peddada L, Heldebrant C, Smith R, Conrad A, Kleinman SH, Busch MP (2003). Dynamics of HIV viremia and antibody seroconversion in plasma donors: implications for diagnosis and staging of primary HIV infection. AIDS.

[74] Beer L, Oster AM, Mattson CL, Skarbinski J, Medical Monitoring Project (2014). Disparities in HIV transmission risk among HIV-infected black and white men who have sex with men, United States, 2009. AIDS.

[75] Blair JM, Fagan JL, Frazier EL, Do A, Bradley H, Valverde EE, McNaghten A, Beer L, Zhang S, Huang P, Mattson CL, Freedman MS, Johnson CH, Sanders CC, Spruit-McGoff KE, Heffelfinger JD, Skarbinski J, National Center for HIV/AIDS‚ Viral Hepatitis‚ STD‚TB Prevention‚ CDC (2014). Behavioral and clinical characteristics of persons receiving medical care for HIV infection - Medical Monitoring Project, United States, 2009. MMWR Suppl.

[76] Centers for Disease Control and Prevention (2015). Medical Monitoring Project.

[77] Centers for Disease Control and Prevention (2015). HIV Surveillance Report.

[78] Hall EW, Sanchez TH, Stein AD, Stephenson R, Zlotorzynska M, Sineath RC, Sullivan PS (2017). Use of videos improves informed consent comprehension in web-based surveys among internet-using men who have sex with men: a randomized controlled trial. J Med Internet Res.

[79] Centers for Disease Control and Prevention (2014). HIV Surveillance Special Report 8.

[80] Finlayson TJ, Le B, Smith A, Bowles K, Cribbin M, Miles I, Oster AM, Martin T, Edwards A, Dinenno E, Centers for Disease ControlPrevention (CDC) (2011). HIV risk, prevention, and testing behaviors among men who have sex with men--National HIV Behavioral Surveillance System, 21 U.S. cities, United States, 2008. MMWR Surveill Summ.

[81] Washington SOOFM Accessed 26 May.

[82] Marmor M, Sheppard HW, Donnell D, Bozeman S, Celum C, Buchbinder S, Koblin B, Seage GR, HIV Network for Prevention Trials Vaccine Preparedness Protocol Team (2001). Homozygous and heterozygous CCR5-Delta32 genotypes are associated with resistance to HIV infection. J Acquir Immune Defic Syndr.

[83] Centers for Disease Control and Prevention HIV Surveillance Special Report 15.

[84] Katz D, Golden M, Stekler J (2012). 2012 National Summit on HIV and Viral Hepatitis Diagnosis. Prevention, and Access to Care.

